# Delayed diagnosis of a primary diffuse large B-cell lymphoma of the humeral head, presenting as pathological fracture: a case report and review of the literature

**DOI:** 10.1016/j.xrrt.2023.12.008

**Published:** 2024-01-26

**Authors:** Andreas Panagopoulos, Konstantina Solou, Argiris Symeonidis, Evgenia Verigou, Olga Kouroukli, Vasiliki Zolota, Zinon T. Kokkalis

**Affiliations:** a Department of Orthopaedics, University Hospital of Patras, Medical School, Patras, Greece; b Hematology Division, Department of Internal Medicine, University Hospital of Patras, Medical School, Patras, Greece; c Department of Pathology, University Hospital of Patras, Medical School, Patras, Greece

**Keywords:** Primary bone lymphoma, Diffuse large B-cell lymphoma, Shoulder, Proximal humerus, Pathological fracture, Reverse shoulder arthroplasty, Chemotherapy, Radiotherapy

Primary lymphoma of the bone (PLB) is a rare lymphoproliferative disorder that comprises 3-5% of primary bone tumors and approximately 5-7% of the extranodal non-Hodgkin lymphomas (NHLs).^14^^,^^38^ It is a malignant lymphoid neoplasm affecting a solitary bone or multiple skeletal sites without visceral or nodal involvement (except from regional lymph nodes).^9^ Diffuse large B-cell lymphoma (DLBCL) is the most common histologic subtype, accounting for up to 80% of cases, affects mainly the axial skeleton, and is more commonly seen in men.^13^^,^^42^ A regional survey among 1225 patients with all types of NHLs, diagnosed between 1991 and 2018 in the area of Western Greece, revealed 32 (2.6%) patients with PLB; the median age was 62 years old, the male/female ratio was 1.43, and 55% of them were <65 years at initial presentation.^40^ PLB can affect any bone, although there is a favor toward bones with persistent bone marrow.^34^ Others suggest that long and flat bones are affected equally.^28^^,^^32^ The most commonly affected bone is femur (29%), followed by pelvis (19%), humerus (13%), head-neck (11%), and tibia (10%).^15^ The monostotic subtype of DLBCL occurs commonly in femur or humerus, while the polyostotic subtype is presented with paraparesis due to vertebral involvement.^21^ Given the rarity of PLB, there is a paucity of high-quality studies to guide treatment, which is usually accomplished with multiagent chemoimmunotherapy with or without radiotherapy.^22^^,^^36^^,^^42^ In most of the cases, there is no place for surgery, except for biopsy and internal fixation or replacement of pathological fractures.^6^^,^^17^^,^^37^ In this article, we report the case of a misdiagnosed DLBCL in a 52-year-old male patient presented to our department with pathological fracture of the humeral head 3 years after his initial complaints. A systematic review of similar cases in the literature is provided as well.

## Case presentation

**Figure 1:**
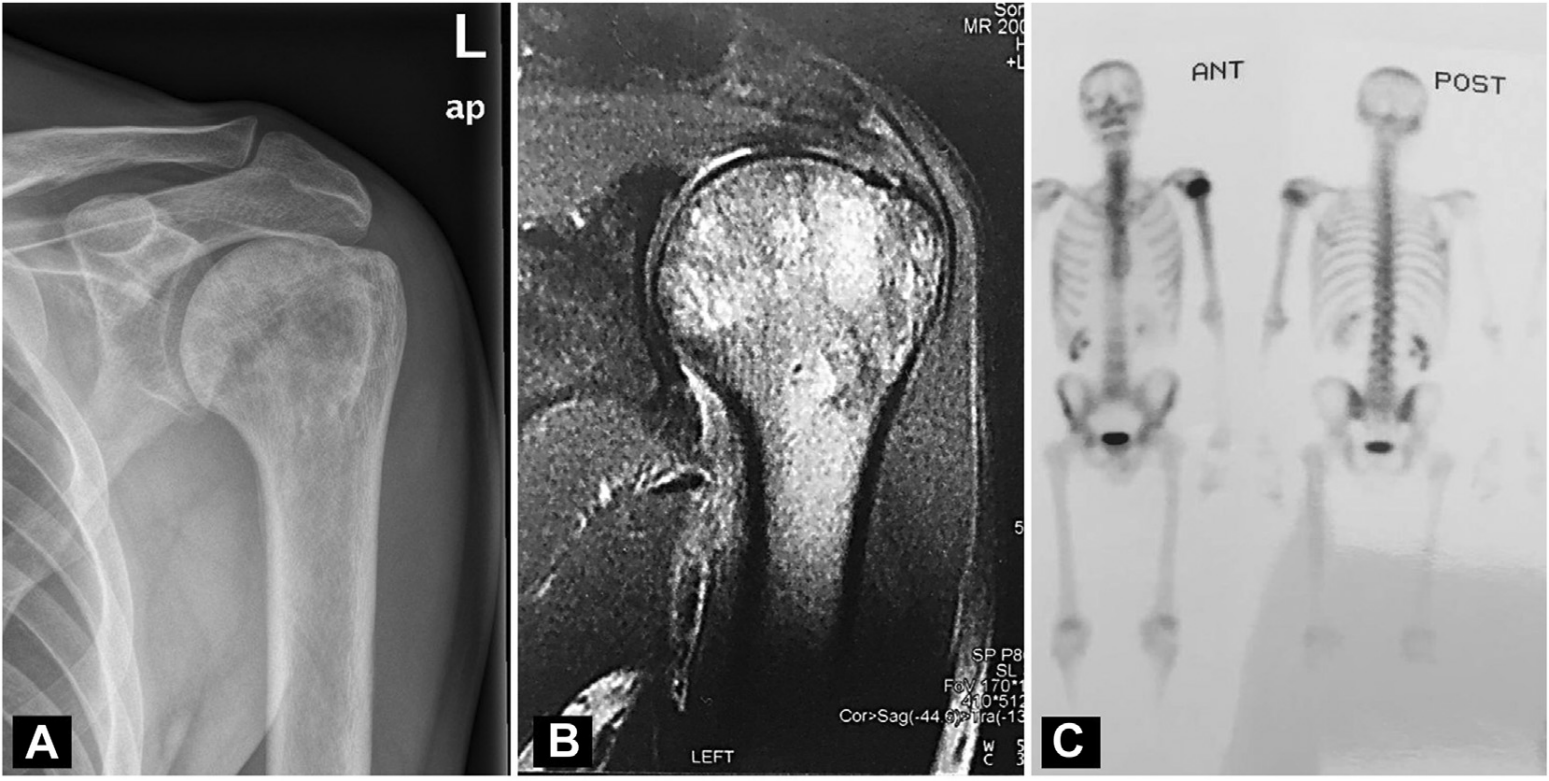
(**A**) Plain X-ray of the left shoulder showing a permeative blastic-sclerotic pattern within the humeral head without cortical thickening and/or periosteal reaction, (**B**) MRI of the shoulder showing the same blastic-sclerotic pattern and some narrowing of the subacromial space, raising the possibility of rotator cuff impingement, (**C**) 3-phase bone scan showing significant uptake in the left proximal humerus. *MRI*, magnetic resonance imaging.

This is a case of a 52-year-old Caucasian man with an unremarkable medical history who presented to the outpatient office in October 2013 with left shoulder pain of a 1-week duration. A written informed consent was taken by the patient for publication purposes including the use of radiological imaging and intraoperative photos. The patient reported that pain started acutely, after a period of heavy working as a barber. He described the pain to be continuous during arm elevation beyond 90°, progressively worsening in the night, non-radiating, and with an intensity of 7/10 on the numerical pain severity scale. He denied having fever, arm weakness, and any tingling or numbness in his left upper extremity. The patient had no past history of a similar complaint, infection, or immunosuppression. On clinical evaluation, he had normal vital signs, absence of high temperature, and pain during anterior elevation implicating signs of subacromial impingement syndrome. The acromioclavicular joint was mildly tender but non-fluctuant. A strength of 4/5 was demonstrated in the muscle groups around the shoulder, elbow, and wrist joints. The age-adjusted Constant score was 87/100. The remainder of the systemic examination was negative for additional pertinent findings, as well as his full laboratory tests, except for an elevated erythrocyte sedimentation rate (33 mm/h). Radiographic examination of the left shoulder revealed a permeative blastic-sclerotic pattern within the humeral head without cortical thickening and/or periosteal reaction. There was some narrowing of the subacromial space, raising the possibility of rotator cuff impingement (Fig. 1, *A*). The patient underwent further radiological investigation with contrast-enhanced magnetic resonance imaging (MRI) and 3-phase bone scan (Fig. 1, *B* and *C*); both exams suggested evidence of possible underlying bone marrow disease and the patient underwent further hematological investigation which provided unremarkable findings.

Finally, a fine-needle bone biopsy was nondiagnostic, mainly showing an infiltrate with extensive crushing artifact and nonspecific immunoexpression. The pathologists suggested a repeated open biopsy but as the patient had significant clinical improvement with analgesics and physiotherapy, he refused to undergo any further investigation.

**Figure 2:**
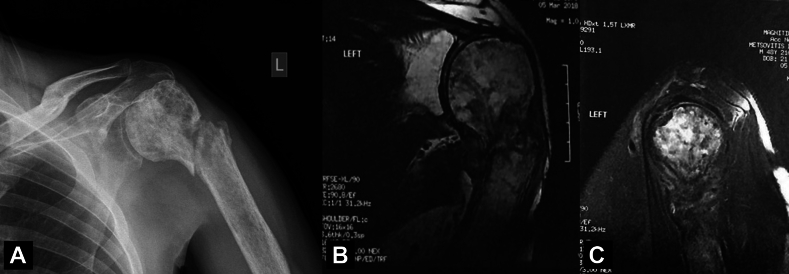
(**A**) Plain X-ray showing a pathological fracture of the humeral head with diffuse permeative sclerotic areas and signs of osteonecrosis, (**B, C**) the same findings are seen in the sagittal and coronal MRI. *MRI*, magnetic resonance imaging.

Three years later (March 2016) he was presented again in the emergency department after experiencing of a sudden and intense pain in his left shoulder, while trying to carry a heavy load. He was unable to move his arm whatever he hold it in a flexed and internally rotated position. On clinical examination, he was afebrile, with no edema, redness or palpable lymph nodes. During the last 3 years, he experienced some discomfort and mild pain when he moved his shoulder beyond 160^°^, but he has not sought for medical care. A radiological examination revealed the presence of a pathological fracture of the humeral head at the surgical neck, with more diffuse permeative sclerotic areas and signs of osteonecrosis (Fig. 2, *A*), a finding that was also confirmed by an MRI study (Fig. 2, *B* and *C*).

**Figure 3:**
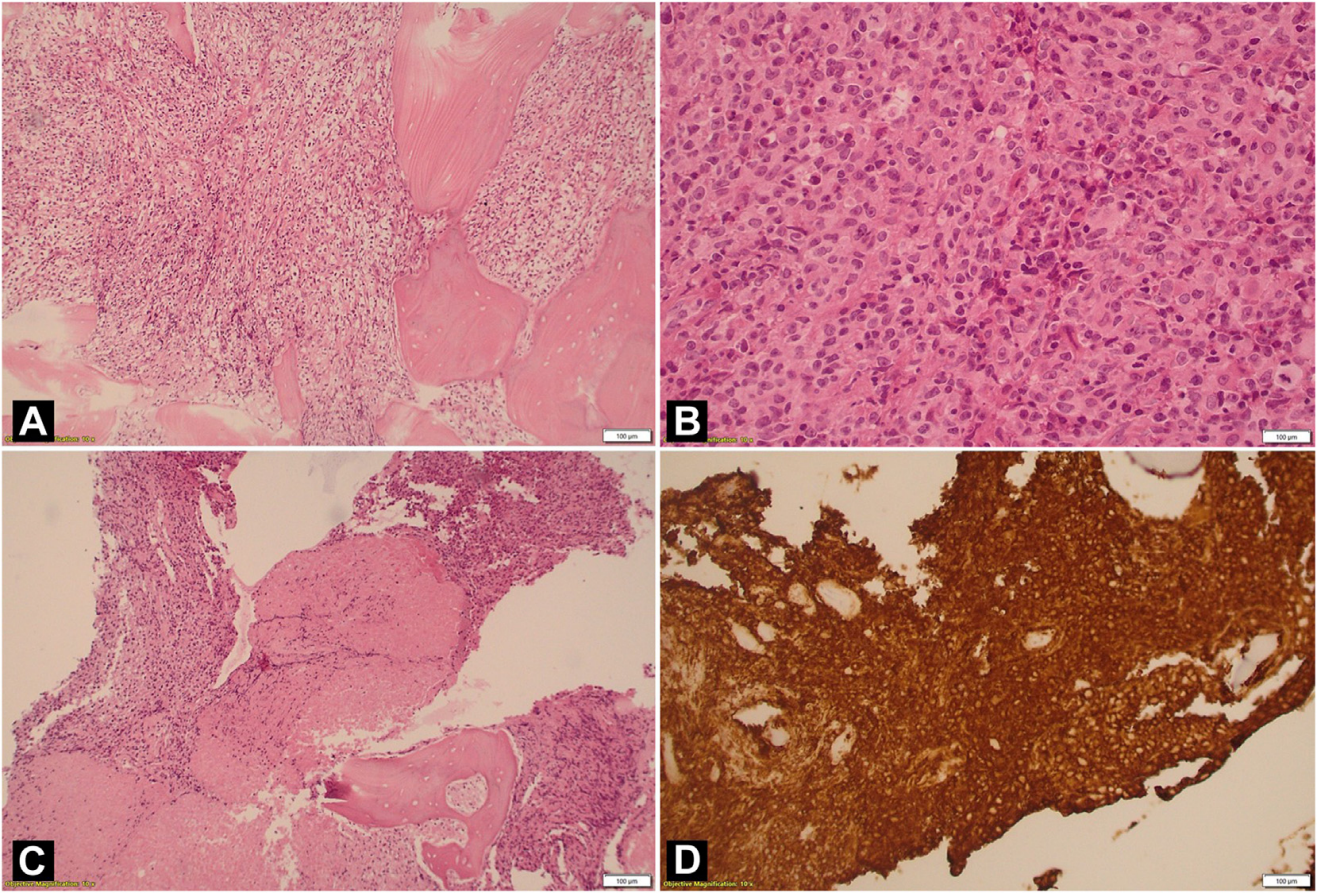
(**A, B**) Histological examination showing a neoplastic population of large, lymphoid cells with clear or pale cytoplasm and ovoid or multilobated nuclei with central nucleoli, (**C**) Areas of extensive coagulative necrosis, (**D**) Immunohistochemically, the neoplastic cells were positive for LCA (3+), CD20 (3+) (Fig. 3D), PAX-5 (3+), CD79a (2+), CD10 (1+), and BCL-6 (1+) and the proliferation rate was around 25%.

The patient was admitted in the hospital and underwent open biopsy of the humeral head. This time, histologic examination revealed diffuse infiltration of the intertrabecular spaces by a neoplastic population of large, lymphoid cells with clear or pale cytoplasm and ovoid or multilobated nuclei with central nucleoli (Fig. 3, *A-C*). The diagnosis of DLBCL of germinal center B-cell-like (GCB) subtype was confirmed.

The oncologic council suggested initially conservative management for the pathological fracture and the patient was left under the care of hematologists for complete staging and antineoplastic treatment. The disease was finally characterized as primary osseous diffuse large B-cell non-Hodgkin’s lymphoma not otherwise specified (NOS), Ann-Arbor stage IEA. He was treated with 8 cycles of chemoimmunotherapy using the RCHOP regimen (rituximab, cyclophosphamide, Adriamycin, vincristine, and methylprednisolone), followed by adjuvant radiotherapy. All treatment courses were well tolerated and uneventful, with occasional administration of preemptive granulocyte colony stimulating factor. He achieved a complete remission, which was confirmed by a positron emission tomography/computed tomography (PET-CT) scan and repeated simple CT scans performed at the scheduled intervals. Complete remission was maintained the latest time the patient was examined in the hematology outpatient clinic on January 2017, that is, 74 months following initial diagnosis or 65 months after the end of his first-line treatment.

**Figure 4:**
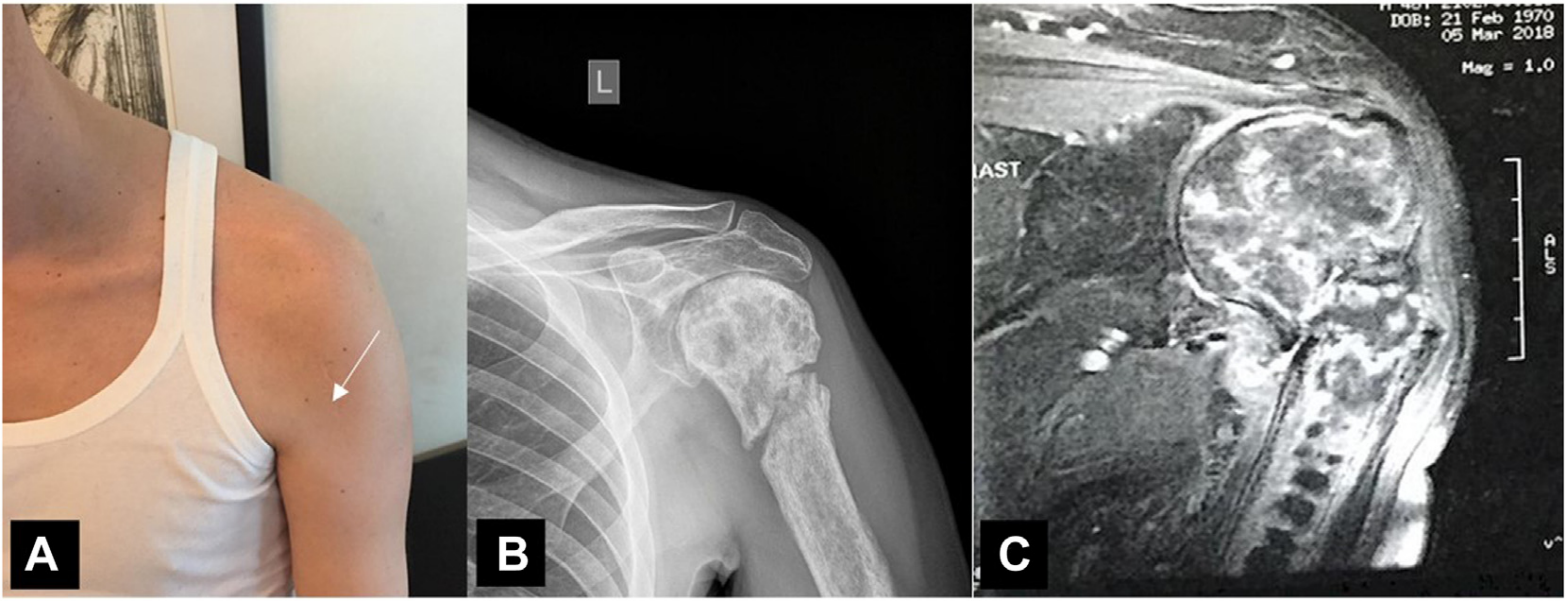
(**A**) Severe muscle atrophy of the anterior (*arrow*) and middle deltoid, (**B, C**) Plain X-ray and MRI scan showing extensive avascular necrosis and signs of postradiation osteoarthritis, along with nonunion of the fracture at the surgical neck and also severe tendinopathy and atrophy of the muscles of the rotator cuff. *MRI*, magnetic resonance imaging.

One year after treatment completion (January 2018), the patient was admitted in the orthopedic department for surgical reconstruction of his left shoulder. Clinically, he was unable to elevate his shoulder beyond 40^ο^ with restricted internal and external rotation and severe muscle atrophy (Fig. 4, *A*). His preoperative Constant score was 55/100. A new radiography and MRI scan (Fig. 4, *B* and *C*) showed extensive avascular necrosis and signs of postradiation osteoarthritis, along with nonunion of the fracture at the surgical neck and also severe tendinopathy and atrophy of the muscles of the rotator cuff; the anterior and middle deltoid muscle was also atrophic.

**Figure 5:**
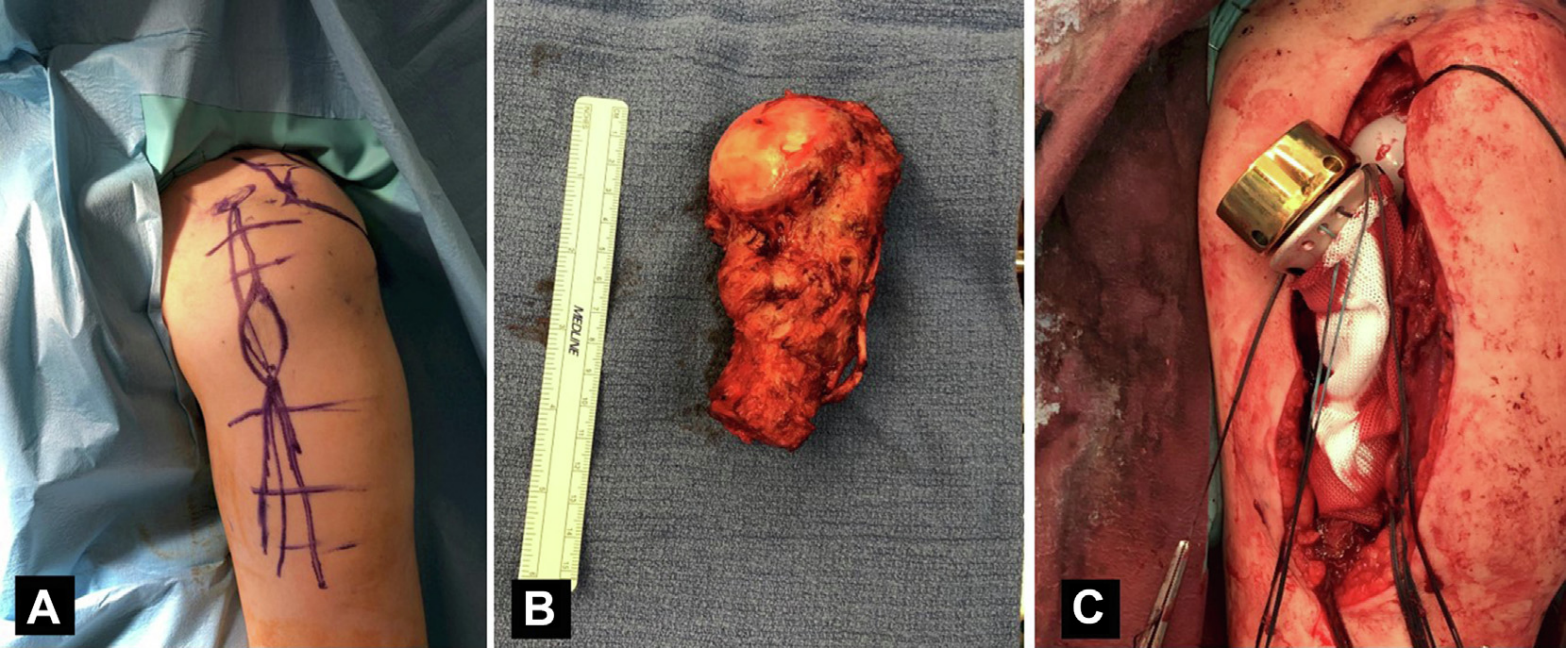
Intraoperative photos showing (**A**) the skin incision with marking of the previous biopsy site, (**B**) the specimen, and (**C**) the mega-prosthesis in place with the appropriate synthetic mesh for capsule and muscle reattachment.

**Figure 6:**
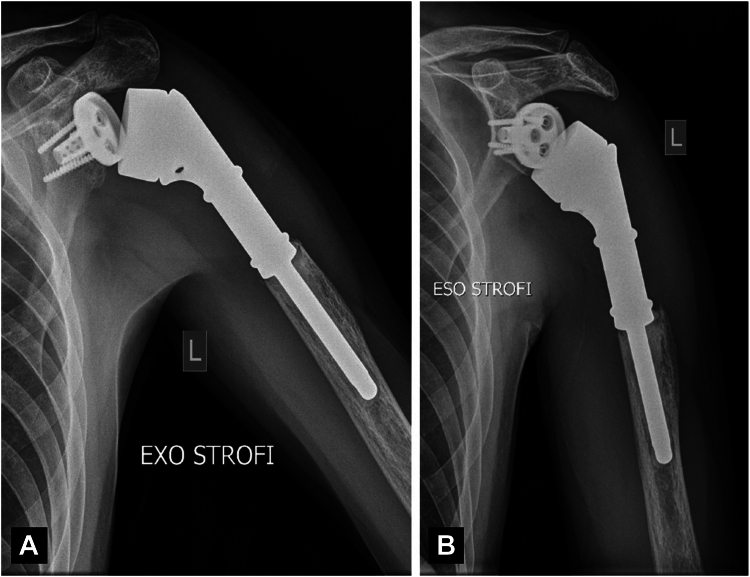
(**A, B**) Follow-up X-rays at 6 months postoperatively, showing normal alignment of the prosthesis without evidence of loosening

The patient consented for total resection of the upper part of the proximal humerus and surgical reconstruction with a tumor-specific reverse shoulder mega-prosthesis (MUTARS Proximal Humeral system; Implantcast GmbH, Buxtehude, Germany). At surgery, we found excessive scarring and atrophy of the deltoid and rotator cuff muscles, necrosis and sclerosis of the humeral head, and no involvement of the glenoid and surrounding tissues. Approximately 12 cm of the proximal part of the humerus were resected and a reverse cemented tumor mega-prosthesis was applied. A tube of synthetic mesh was used for capsule and muscle reattachment (Fig. 5). The postoperative course was uneventful, and the patient followed a special rehabilitation program, being able to regain a functional range of motion at 6 months postoperatively (150^°^ anterior elevation, 45^°^ internal rotation, and 40^°^ external rotation). He had no pain and was able to perform most of his daily living activities. The Constant score was 77 and the radiological examination of the shoulder was unremarkable (Fig. 6). As previously pointed out, there was not any evidence of local recurrence or systemic dissemination of the basic disease (PET/CT, conventional CT).

**Figure 7:**
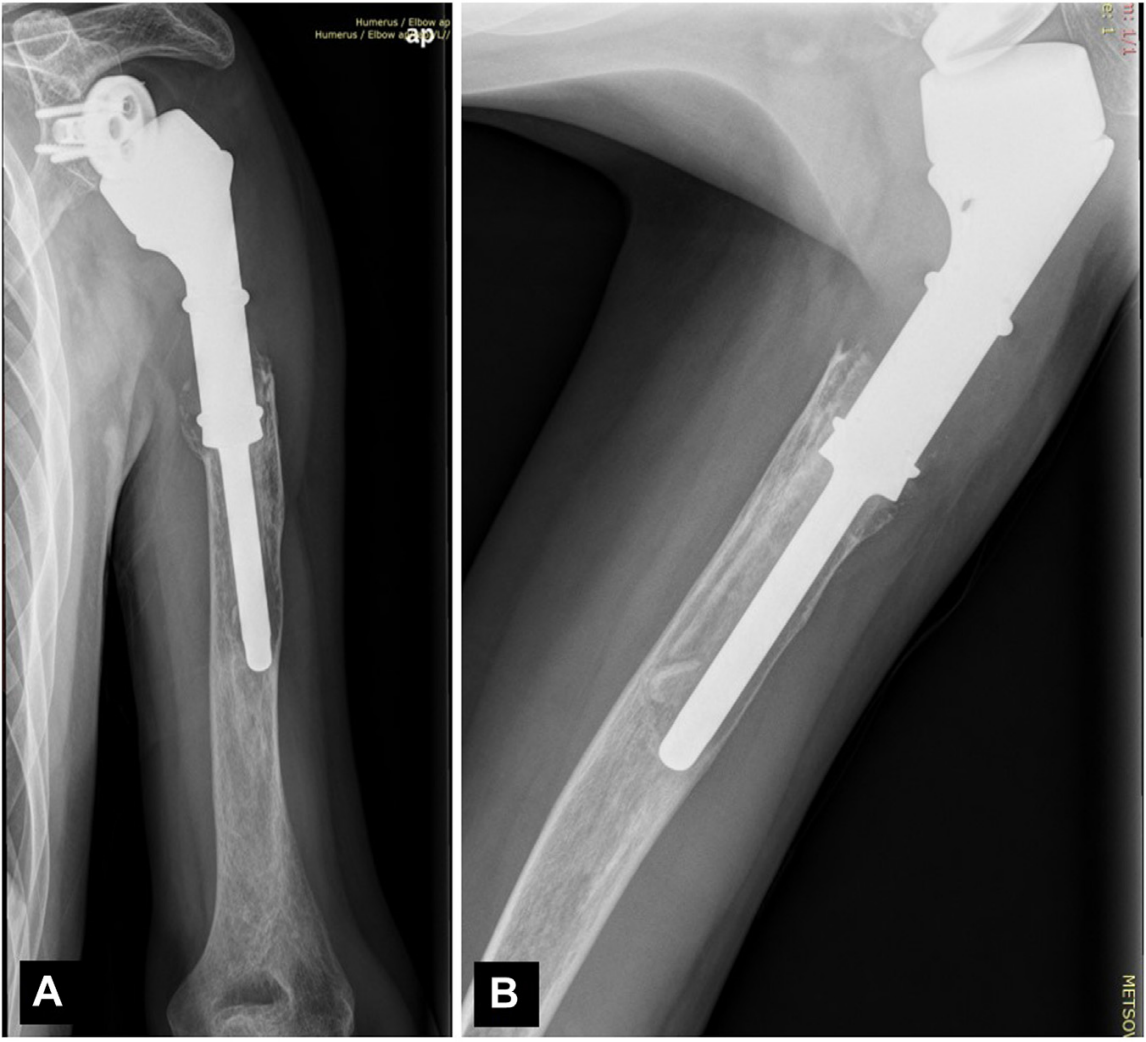
(**A, B**) Follow-up X-rays 2 years later, showing osteolysis of the proximal part of the humerus, loosening and extrusion of the prosthesis toward the lateral cortex.

**Figure 8:**
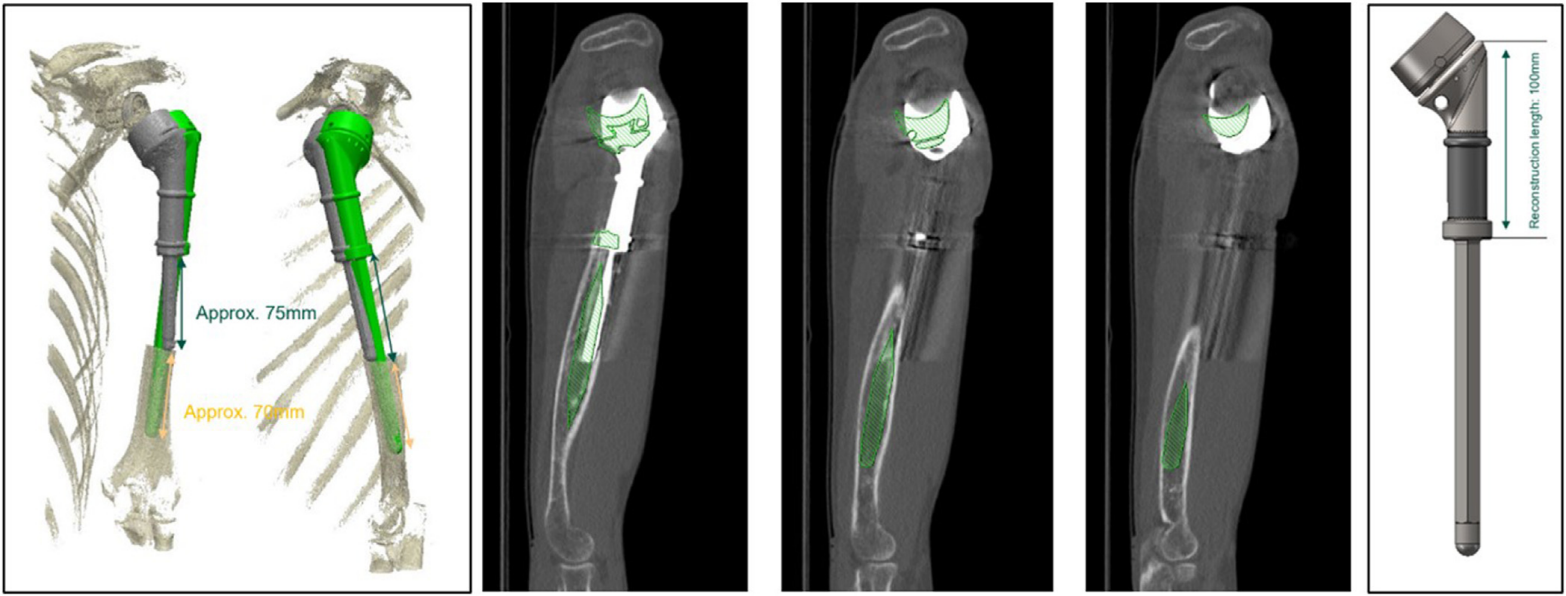
CT reconstruction and preoperative planning for the ordering of a longer custom-made prosthesis. *CT*, computed tomography.

Two years later (December 2020), he presented with severe pain and an audible click in the humerus while moving his shoulder. He was unable to elevate his shoulder beyond 40^°^ and he had very limited internal and external rotation. At clinical examination, there was a marked paradoxical motion at the distal part of the humerus that aggravated his symptoms. A new shoulder radiography showed evidence of loosening, osteolysis, and prosthesis excursion at the lateral cortex (Fig. 7). A revision operation was planned ordering a longer, cemented, custom-made Mutars Humerus stem Ø10X145 mm. For this purpose, a preoperative 3-dimensional CT scan was performed and was sent to the company for appropriate planning (Fig. 8).

**Figure 9:**
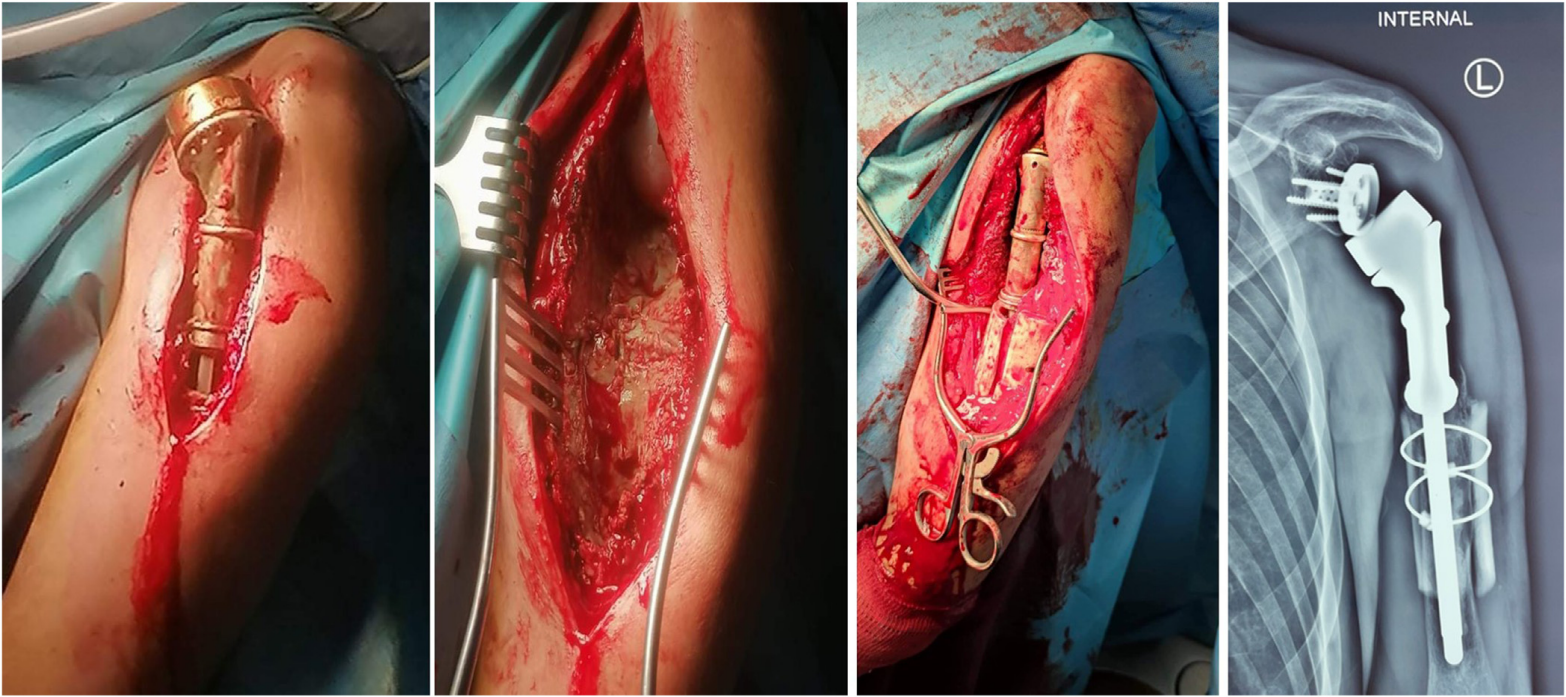
Intraoperative photos of the revision operation and postoperative X-ray showing the longer stem and the splitted allograft.

**Figure 10:**
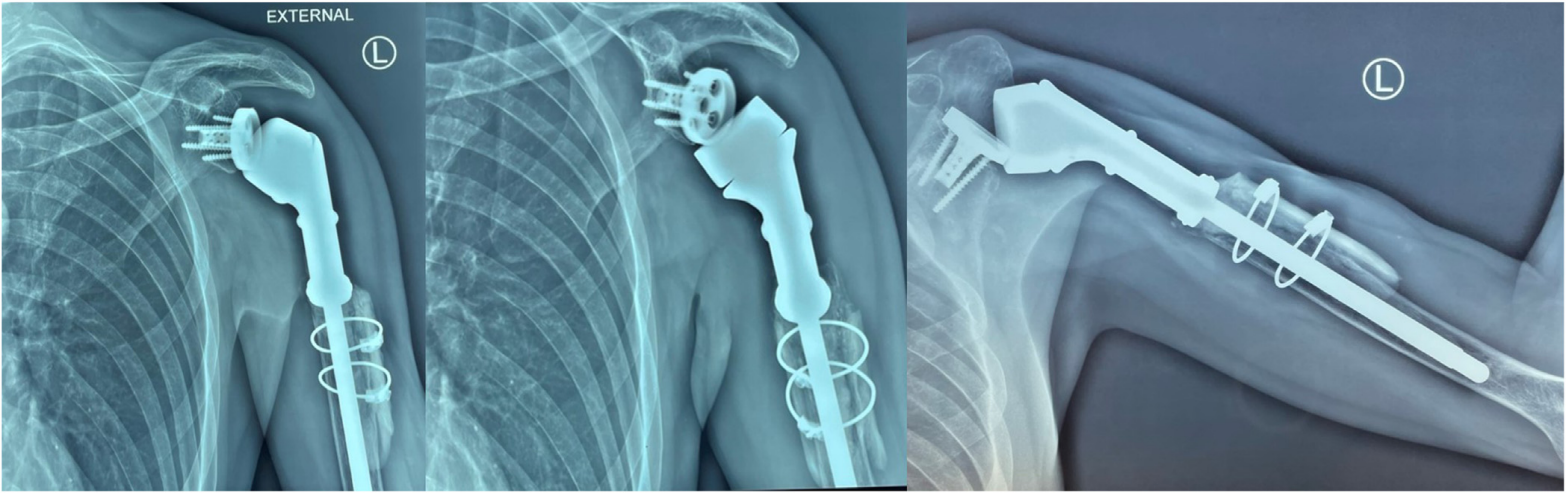
The final radiological examination at 2 years, showing a stable prosthesis, no evidence of loosening and partial allograft incorporation.

At surgery, the old prosthesis was removed first, revealing significant scar tissue formation that was removed meticulously; culture samples taken for different sites were negative for low-grade infection (Fig. 9). The longer Mutars stem was assembled and the prosthesis was reimplanted with cement after minimal reaming. A split femoral allograft was applied at the junction of the prosthesis with the humerus using cerclage wires. The postoperative course was normal, and the patient was prescribed with a less aggressive rehabilitation program. At the 2-year follow-up, he had a Constant score of 82, no pain, and he was back to work. The final radiological examination at 2 years was normal with no evidence of loosening and with partial allograft incorporation (Fig. 10).

## Discussion

Primary bone lymphoma (PBL) is defined as a malignant neoplasm of lymphoid cell that presents with one or more bony lesions without nodal involvement or other extranodal lesions.^7^ PBLs represent NHLs and account for 1% of all lymphomas, 3-7% of extranodal lymphomas, and 7% of malignant primary bone tumors; the DLBCL is by far the most common histological type representing over 80% of all cases.^15^^,^^16^^,^^41^ PBL may occur at all ages, with a typical diagnosis age of 45–60 years.^18^^,^^38^ The risk and pathogenetic factors contributing to the development of DLBCL include immunosuppressive medication used in transplant patients, family or personal history of lymphoma, history of radiation, chemical agents such as dyes and pesticides, as well as white race and obesity.^8^^,^^23^ Our patient had a negative family history and no other predisposing factors for lymphoma.

The clinical features of DLBCL are generally nonspecific, frequently leading to a delay in diagnosis, as in our case. The most common symptom of PBL is an insidious, intermittent, and progressively worsening local bone pain in the affected area, which is not relieved by rest and medication.^4^^,^^6^^,^^10^^,^^18^^,^^26^^,^^29^ Other common manifestations include soft tissue edema, palpable mass, pathological fracture, restricted range of motion, and rarely the typical “B” symptoms^18^^,^^26^^,^^29^^,^^35^^,^^37^^,^^38^ (fever, night sweats, and weight loss). Cases with a history of occult trauma in the affected area have also been reported^19^^,^^39^ as well as misdiagnosed cases initially treated for tendinopathy/bursitis in the shoulder^3^^,^^31^ or muscle spasm in the neck.^12^ DLBCL can develop in any part of the skeleton, although is most common in the femur, humerus, tibia, spine, and pelvis. Other less common sites of occurrence include the skull, forearm, scapula, clavicle, patella, hands, and feet.^4^^,^^15^^,^^32^^,^^37^

The imaging appearance of DLBCL is highly variable and unspecific but it is crucial for the initial depiction and lesion extent, guidance of biopsy, staging, and restaging as well as monitoring of the treatment response. Initial X-rays may be equivocal, frequently leading to a diagnostic delay.^30^ Krishnan et al^20^ reported 3 radiographic patterns in plain X-rays that PBL can manifest: (a) the lytic-destructive pattern; (b) the blastic-sclerotic pattern; and (c) the subtle or “near-normal” findings where conventional X-rays fail to depict any notable finding. In our case, the radiological pattern was of permeative blastic-sclerotic type without cortical thickening or periosteal reaction, but initial biopsy did not suggest DLBCL at first place. Kishan Prasad et al^19^ reported a similar case in a 31-year-old male patient who presented with pain and swelling in his right shoulder for the last 5 months. The plain X-ray showed complete destruction of the upper end of the humerus, although a previous one taken 6 months ago had shown only osteoporotic changes in the humeral head. The authors successfully made a diagnosis of DLBCL using fine needle aspiration cytology. CT is the primary modality for radiologically guided biopsy and can effectively depict soft tissue extension, bone marrow involvement, cortical disruption, and pathological fractures; in addition, CT can be used for the staging, restaging, and follow-up.^4^^,^^16^ MRI is the modality of choice for the early detection of PBL and the depiction of its soft tissue extension and bone marrow involvement; the lesion most commonly appears hypointense in T1-weighted images and hyperintense in T2-weighted images, while areas of enhancement within the neoplasm can be demonstrated after gadolinium administration.^11^^,^^16^ The main drawback of MRI remains its low specificity when restaging, and for this reason, the 18-fluorodeoxyglycose PET-CT has been proposed as the standard care for staging, restaging, surveillance of recurrence, and monitoring of treatment response.^11^^,^^25^ Bone scan is considered a standard imaging tool in the diagnostic workup of musculoskeletal tumors, however, it has been shown to present a lower sensitivity and specificity than 18-fluorodeoxyglycose/PET in detecting lymphomatous infiltration of bone.^4^ Sakurada et al^33^ were able to accidentally detect a DLBCL in the shoulder of a 77-year-old patient who was investigated for other reasons with cardiac ^123^I-MIBG scintigraphy.

The definite diagnosis of PBL is histological with immunohistochemical examination; tissue samples can be obtained either via image-guided percutaneous fine-needle, core-needle, or open biopsy with the core-needle being the preferred method.^4^ Diffuse large B-cell lymphoma (DLBCL) is the predominant histological type, constituting approximately 80% of cases.^16^^,^^32^ DLBCL is divided histologically into two subtypes: a GCB phenotype (CD10 +, BCL-6 +, MUM-1 +) and a nongerminal center phenotype (CD10 -, BCL-6 -, MUM-1 +).^1^^,^^16^^,^^32^ Our patient’s neoplastic cells were positive for LCA, CD20, PAX-5, CD79a, CD10, and BCL-6, so it was considered a GCB subtype.

Treatment of PBL is based on systemic therapy including chemotherapy or immunochemotherapy with or without radiotherapy, resulting in a 5-year overall survival of approximately 70-80%.^16^^,^^30^ Anthracycline-based, multiagent chemotherapy comprising cyclophosphamide, doxorubicin, vincristine, and prednisone (CHOP) with or without the addition of rituximab (R-CHOP) is the preferred modality.^32^^,^^37^ Bruno Ventre et al^5^ claimed that chemotherapy is more effective than radiotherapy in DLBCL-NOS cases but Beal et al^2^ in contrast, found that PBL patients managed with a combined regimen (chemotherapy and radiotherapy) versus a single modality therapy had a significantly superior outcome, with a 5-year overall survival of 95 and 78%, respectively. Hence, chemotherapy and radiotherapy are usually combined, as in our case. The role of surgery is generally limited and it is used for diagnostic purposes (biopsy), fixation of impeding or acute pathological fractures, as in our case, neurological complications (spinal cord compression), segmental defects in long bones, and destruction or articular collapse caused by avascular necrosis following treatment.^5^^,^^17^^,^^37^ Scoccianti et al^37^ reported a 19% rate of surgery other than biopsy in patients affected only by pathological fractures; a similar rate of surgery was reported by Marshall et al^27^ (17.8%) and Jawad et al^15^ (26.3%) in a wide multicenter database of 1500 PBLs.

Our systematic literature review for cases of primary DLBCL in the shoulder region revealed 14 reported cases so far^3^^,^^6^^,^^12^^,^^18^^,^^19^^,^^26^^,^^29^^,^^31^^,^^35^^,^^37^, ^38^, ^39^ (Table I). There were 9 males and 5 females with a mean age of 48 years old (range, 15-80 years). The main clinical symptom was pain, with an average duration of 5.5 months (range, 2.5-12 months) prior to definite diagnosis; 7 patients (50%) presented with soft tissue mass, 2 with marked swelling, 3 had concomitant lymphadenopathy, 2 presented with pathological fracture, and only one with typical B-symptoms and pathological fractures. All had severe bony destruction of the proximal humerus in the subsequent radiological investigation. One report had no information about treatment regimen; for the rest cases, combined chemotherapy and radiotherapy were administered in 7 (50%) patients, chemotherapy alone in 4 and radiotherapy alone in one. One patient showed intolerance in chemotherapy and stopped his treatment in an early face. In four reports, there was no information regarding outcome; in the rest 11 cases, there was no evidence of recurrence in 10/11 patients in a follow-up period ranging from 2 months to 17 years. Surgery was performed in two patients: Scoccianti et al^37^ reported a case series of 21 patients with DLBCL; they applied a humeral megaprosthesis in a 23-year-old female patient with destruction of the humeral head after the completion of combined chemotherapy (CHOP), radiotherapy, and autologous bone marrow transplant. The patient, similar to our case, had no signs of recurrence after 88 months of follow-up. Caporale et al^6^ reported an 80-year-old male patient presenting with a six-month history of continuous severe pain to the right shoulder. MRI showed an ingrowing inhomogeneous osteolytic lesion in the anteromedial aspect of the right humeral head. He underwent shoulder arthroscopy, excluding intra-articular involvement, and simultaneously percutaneous bone biopsy diagnostic for lymphoma. Three days later, he underwent surgical excision of the mass and implantation of a reverse shoulder prosthesis. The patient started chemotherapy according to the CHOP protocol but did not tolerate it because of the sudden onset of herpes zoster. At 9-month follow-up, he had no shoulder pain and no evidence of local or systemic recurrence. Regarding the loosening in our case, 2 years postoperatively, we believe that the main reason was the short length of the distal part of the megaprosthesis considering the large amount of proximal humerus that has been resected at first place. As the patient regained his range of motion and used his shoulders above 90^°^ working as a barber, this allowed micromotion at the junction between the bone and the prosthesis leading gradually to asepting loosening, extrusion of the distal part, and obliteration of the lateral cortex. A longer prosthesis should have been used from the beginning.

**Table I:** Cases of reported DLBCL in the shoulder.

Study	Year	Age	Gender M/F	Symptoms/radiological examination	Image	Therapy	Last follow-up-metastasis
Stemberga et al^39^	2003	21	M	Injury in the right shoulder 10 m ago with clear X-rays, severe pain thereafter, destruction of humeral head in X-rays		Radio (35Gy)	17y/no
Siddiqui et al^38^	2013	23	M	Pain and swelling in the left shoulder for 3 m **Pathological fracture**		Radio (25Gy), 6 cycles CHOP	12m/no
Kishan Prashad et al^19^	2013	31	F	Trivial trauma 4 m ago in the right shoulder Pain and swelling for 5m, destruction of humeral head in X-rays		Radio (50Gy) 6 cycles CHOP	3y/no
Mondello et al^29^	2014	78	M	Severe pain for 4 mo, large humeral mass and concomitant axillary lymphadenopathy		Radioimmunotherapy (90Y-IT) 6 cycles R-CHOP	1y/first relapse 3 more relapses
Scoccianti **e**t al^37^	2013	23	F	Severe bone destruction in right proximal humerus No clinical information was provided		Radio (NR) CHOP + ABTM Megaprothesis after treatment	88 m/no
Liu YC et al^24^	2012	53	F	One-year progressive pain, subclavian/axillary lymphadenopathy, destruction of proximal humerus		6 cycles R-CEOP	NR
Liu SZ et al^26^	2018	64	F	6 months progressive pain, rapidly increasing mass reaching (15.0 cm × 15.0 cm) after 2 mo, destruction of the humeral head		8 cycles R-CHOP Radio (50Gy)	2y/no
Daliparty et al^10^	2021	31	M	Progressively worsening pain in the left shoulder, swelling and warmth, infiltrative bone marrow abnormality with large soft tissue masses		8 cycles R-CHOP	2m normal PET
Hatem J & Bogusz AM^12^	2016	79	M	Neck pain initially, painless mass 2.5 months later in the shoulder, (12.5 × 4.5 × 2.5 cm) infiltrating the lateral head of the triceps with marrow replacing lesions involving the humeral		5 cycles R-CHOP (refused the 6^th^)	Complete remission after the 5th cycle (PET)
Khan AQ et al^18^	2023	15	M	Diffuse joint pain in his body for 6 months with recent worsening in his left shoulder and elbow B-symptoms, fracture of the humeral head and intercondylar region, expansile lytic lesions		Under palliative chemotherapy	NR
Pitman et al^31^	2003	70	F	Intensive pain in the left shoulder (treated as bursitis) for 5 months, 9-cm mass eroding into the left scapula		NR	NR
Beutler et al^3^	2019	51	M	Pain in the left shoulder for 4 months, treated as rotator cuff tendinitis, testicular swelling, large tumor replacing the marrow of the proximal humerus and nodal mass in the left axilla (6.4 × 7.5 × 2.8 cm)		6 cycles R-CHOP Radio (35Gy)	NR
Sarma YS & Sriharibabu M^35^	2019	53	M	Pain and swelling of 3 months duration over the posterior aspect of the right shoulder, large soft tissue mass with permeative destruction of the right scapula and enlarged axillary and pectoral lymph nodes		6 cycles R-CHOP Radio (39.5 Gy)	18m/no
Caporale et al^6^	2013	80	M	6-month history of continuous severe pain to the right shoulder, infiltration, osteolysis of the cortical bone, bone marrow involvement, he underwent surgical excision and application of reverse shoulder prosthesis		No tolerated CHOP because he developed herpes zoster	9m/no

*DLBCL*, diffuse large B-cell lymphoma; *Radio*, radiotherapy; *CHOP*, cyclophosphamide, doxorubicine, vincristine, prednisone; *R-CHOP*, rituximab, cyclophosphamide, doxorubicine, vincristine, prednisone; *90Y-IT*, Y-ibritumomab tiuxetan; *ABMT*, autologous bone marrow transplant; *R-CEOP*, rituximab, cyclophosphamide, epirubicin, vincristine, and prednisolone; *PET*, positron emission tomography; *B-symptoms*, intermitted fever, night sweats, and weight loss.

## Conclusion

PBLs are rare malignant neoplasms of lymphoid origin that affect one or more bones without nodal involvement or other extranodal lesions; the DLBCL, is by far the most common histological type representing over 80% of all cases. Patients with PBL in the shoulder are usually present with chronic pain, palpable mass, or pathological fractures. MRI and PET-CT are important for initial diagnosis, but this must be established with bone biopsy and immunocytochemical studies. Chemotherapy and radiotherapy can provide complete regression in the majority of the patients; surgical treatment is used in neglected cases to obtain biopsies and when there is severe functional impairment. Reverse shoulder megaprosthesis with or without allograft may benefit the patients with severe impairment.

## Disclaimers:

Funding: No funding was disclosed by the authors.

Conflicts of interest: The authors, their immediate families, and any research foundation with which they are affiliated have not received any financial payments or other benefits from any commercial entity related to the subject of this article.

Patient consent: Obtained.
